# Efficacy of Different Nitric Oxide-Based Strategies in Preventing Experimental Cerebral Malaria by *Plasmodium berghei* ANKA

**DOI:** 10.1371/journal.pone.0032048

**Published:** 2012-02-13

**Authors:** Yuri C. Martins, Graziela M. Zanini, John A. Frangos, Leonardo J. M. Carvalho

**Affiliations:** 1 Center for Malaria Research, La Jolla Bioengineering Institute, San Diego, California, United States of America; 2 Laboratory of Inflammation and Immunity, Institute of Microbiology, Federal University of Rio de Janeiro, Rio de Janeiro, Brazil; 3 Parasitology Service, Evandro Chagas Clinical Research Institute, Fiocruz, Rio de Janeiro, Brazil; 4 Laboratory of Malaria Research, Oswaldo Cruz Institute, Fiocruz, Rio de Janeiro, Brazil; Université Pierre et Marie Curie, France

## Abstract

**Background:**

Low nitric oxide (NO) bioavailability plays a role in the pathogenesis of human as well as of experimental cerebral malaria (ECM) caused by Plasmodium berghei ANKA (PbA). ECM is partially prevented by administration of the NO-donor dipropylenetriamine NONOate (DPTA-NO) at high concentration (1 mg/mouse), which also induces major side effects such as a sharp drop in blood pressure. We asked whether alternative strategies to improve NO bioavailability with minor side effects would also be effective in preventing ECM.

**Methodology/Principal Findings:**

Mice were infected with PbA and prophylactically treated twice a day with bolus injections of L-arginine, Nω-hydroxy-nor-Arginine (nor-NOHA), tetrahydrobiopterin (BH4), separately or combined, sodium nitrite, sildenafil or sildenafil plus DPTA-NO starting on day 0 of infection. L-arginine and BH4 supplementation, with or without arginase inhibition by nor-NOHA, increased plasma nitrite levels but failed to protect against ECM development. Accordingly, prophylactic treatment with continuous delivery of L-arginine using osmotic pumps also did not improve survival. Similar outcomes were observed with sodium nitrite sildenafil (aimed at inhibiting phosphodiesterase-5) or with DPTA-NO. However, sildenafil (0.1 mg/mouse) in combination with a lower dose (0.1 mg/mouse) of DPTA-NO decreased ECM incidence (82±7.4% mortality in the saline group and 38±10.6% in the treated group; p<0.05). The combined prophylactic therapy did not aggravate anemia, had delayed effects in systolic, diastolic and mean arterial blood pressure and induced lower effects in pulse pressure when compared to DPTA-NO 1 mg/mouse.

**Conclusions/Significance:**

These data show that sildenafil lowers the amount of NO-donor needed to prevent ECM, resulting also in lesser side effects. Prophylactic L-arginine when given in bolus or continuous delivery and bolus BH4 supplementation, with or without arginase inhibition, were able to increase NO bioavailability in PbA-infected mice but failed to decrease ECM incidence in the doses and protocol used.

## Introduction

Human cerebral malaria (HCM) is a life-threatening condition and remains a serious public health problem in a number of tropical and sub-tropical countries [1]. Despite anti-malarial treatment, the disease has a significant mortality rate of 18–30% and a significant proportion of children who survive are left with cognitive disability (∼25%) and neurologic deficits (1.1–4.4%), for which there is often very little long-term support or treatment [2], [3], [4], [5], [6].

The murine model of cerebral malaria induced by Plasmodium berghei ANKA (PbA) in susceptible mouse strains is considered to present a number of similarities with HCM in terms of physiopathogenesis. It is also thought to present a number of differences and therefore there is no consensus to its relevance to HCM [7], [8], [9], [10], [11], [12], [13]. However, low nitric oxide (NO) bioavailability is believed to play a significant role in both HCM and murine or experimental cerebral malaria (ECM). ECM is associated with a microvascular dysfunction in the brain characterized by vasoconstriction, vascular occlusion, endothelial activation with intravascular inflammation, microhemorrhages and eventually vascular collapse [14], [15]. Endothelial dysfunction is at least in part explained by a state of low NO bioavailability in PbA-infected mice that has been argued to result mainly from the low plasma levels of L-arginine [16], the substrate used by the NO synthases (NOS) to generate NO plus citrulline [17], [18], as well as from the NO-scavenging effect of cell-free hemoglobin due to parasite-induced hemolysis [16]. Similarly, patients with severe malaria, including HCM, show low levels of exhaled NO, endothelial dysfunction [19], reduced endothelial NO synthase expression [20], hypoargininemia [19], [21], and increased levels of acellular plasma hemoglobin [22].

Prophylactic treatment of PbA-infected mice with the NO-donor dipropylenetriamine NONOate (DPTA-NO) can partially prevent ECM development [15], [16]. However, DPTA-NO has been shown to ameliorate microvascular dysfunction and prevent ECM development only at high concentrations (1 mg/mouse every 12 hours) with the generation of NO levels well above those attained under physiological conditions [16]. These high levels induce important side effects such as marked hypotension [16] and worsening of the infection-induced anemia [15]. Therefore, It remains to be shown whether more physiologically and clinically relevant strategies to improve NO bioavailability are also effective in preventing ECM development while generating less significant side effects.

Several factors may help to explain the states of low NO bioavailability and hypoargininemia that occur during ECM, consequently a number of interventions can be devised to correct these deficiencies. In addition to releasing hemoglobin, hemolysis may also release large amounts of arginase, which competes with NOS for the same substrate, L-arginine, depleting its endogenous pools and generating urea plus ornithine rather than NO [23], [24]. Arginine supplementation, with or without arginase inhibition, is therefore expected to improve NO bioavailability. Interestingly, although clinical trials with L-arginine infusion have been performed in malaria endemic areas and showed that it ameliorates malaria-related endothelial dysfunction and is safe [19], [25], this approach has not been explored in ECM. On the other hand, even in the presence of proper amounts of substrate, NOS malfunction may occur and result in decreased production of NO. In fact, a decrease of NOS activity in the brain has been shown in PbA-infected moribund mice [26], [27]. NOS is a homodimeric oxidoreductase containing heme, flavin adenine dinucleotide, flavin mononucleotide, and tetrahydrobiopterin (BH4), which is a cofactor essential for the catalytic activity of three major NOS isoforms: neuronal (nNOS), inducible (iNOS), and endothelial (eNOS) [17]. The coupling between the NO substrate, L-arginine, and the heme site requires BH4 to bind in the dimer interface of NOS. BH4 depletion, which results from its oxidation and/or reduced synthesis, causes functional uncoupling of NOS, which results in the generation of more superoxide and less NO [28]. In this case, BH4 supplementation may improve NOS function.

One alternative strategy to overcome the low NO bioavailability in ECM without boosting NO generation is to maximize signaling by the available NO. The most studied and well characterized pathway by which NO can exercise its biological effects is through soluble guanylyl cyclase (sGC) activation, resulting in increased levels of cyclic guanosine monophosphate (cGMP) [17]. cGMP levels are decreased by 60% in the brain of mice with ECM and prophylactic administration of DPTA-NO can restore them to values encountered in uninfected animals [16]. In healthy states, tissue cGMP levels are determined by a balance between the activities of sGC and cyclic nucleotide phosphodiesterases (PDEs) that catalyze the breakdown of cGMP [29]. Drugs such as sildenafil increase brain cGMP levels through inhibition of PDE-5 and therefore prolong the downstream effects of NO, providing for improved NO activity without changing NO generation [30]. Therefore, sildenafil administration alone or coupled with lower doses of NO-donors such as DPTA-NO itself might be expected to magnify the effects of limited endogenous NO production and prevent ECM without causing the marked side effects observed with high doses of DPTA-NO.

Finally, alternative NO donors could be considered. It has been experimentally [31], [32] and clinically [33], [34] shown that deoxygenated hemoglobin in an acidic environment reduces nitrite to NO causing vasodilation. This effect may have an important function in providing NO in sites of low oxygenation, such as in ischemia. Since we have previously shown that ECM is associated with vasospasm, decreased blood flow, and regional hypoperfusion in the brain [14], we anticipated that administration of sodium nitrite to PbA-infected mice might lead to endogenous NO generation and amelioration of the microcirculatory dysfunction decreasing mortality.

In the present study, we evaluated the efficacy and safety of these different strategies, independently or in combination, in preventing the development of ECM and improving NO bioavailability during the infection by P. berghei ANKA. We show that none of the strategies aimed to increase the endogenous production of NO by NOS, or boost its effects through the sGC pathway, were efficient to prevent ECM. We also show that lower doses of DPTA-NO did not prevent ECM, but protection was obtained when this strategy was combined with PDE-5 inhibition by sildenafil. Finally, we show that the combined therapy did not produce all the adverse side effects generated by the prophylactic treatment with high doses of DPTA-NO alone.

## Materials and Methods

### Mice, parasites and infection

This study was carried out in strict accordance with the recommendations in the Guide for the Care and Use of Laboratory Animals of the National Institutes of Health. All experimental protocols were reviewed and approved by LJBI's Institutional Animal Care and Use Committee (Permit Number: NO-Heme 001) and all efforts were made to minimize suffering. Six to eight week old C57BL/6 mice (18–20 g) were obtained from Jackson Laboratories (Bar Harbor, ME). Mice were housed in groups of no more than five per cage with free access to chow and water and allowed to adapt to their new environment for three days before experimentation. The Plasmodium berghei ANKA PbA-GFPcon 259cl2, which is a genetically modified parasite of clone cl15cy1 of the ANKA strain that expresses GFP constitutively during the whole life cycle, was used (a kind donation of MR4, Manassas, VA; deposited by CJ Janse and AP Waters; MR4 reagent number: MRA-865). The parasite was propagated in C57BL/6J mice and in each experiment a fresh blood sample was obtained from a passage mouse and a suspension containing 1×10^6^ parasitized red blood cells (pRBC) in 100 µL was injected intraperitoneally (IP) in each mouse of the experimental groups. Parasitemia was checked using flow cytometry and quantified by counting the number of pRBC in 10,000 RBC beginning on day 4 after infection.

### Clinical assessment

Motor behavior and rectal temperature were checked daily as described before [35]. Briefly, a set of six simple behavioral tests (transfer arousal, locomotor activity, tail elevation, wire maneuver, contact righting reflex and righting in arena) adapted from the SHIRPA protocol [36], [37] was used to provide a better estimate of the overall clinical status of the mice during infection. The performance in each test was assessed and a composite score was determined ranging from 0 to 23, where 23 indicates maximum performance and 0 indicates complete impairment – usually coma. Body temperature was monitored using an Accorn Series Thermocouple thermometer with a mouse rectal probe (Oakton Instruments, Vernon Hills, IL). ECM was defined as the presentation of one or more of the following clinical signs of neurological involvement: ataxia, limb paralysis, poor righting reflex, seizures, roll-over, coma.

### Treatments

PbA-infected mice were treated with either saline, dipropylenetriamine NONOate (DPTA-NO, Cayman Chemical, Ann Arbor, MI – 0.01, 0.1 and 1 mg/mouse), L-arginine (Sigma-Aldrich, St. Louis, MO – 4 mg/mouse), tetrahydro-L-Biopterin (BH4, Cayman Chemical – 1 mg/mouse), Nω-hydroxy-nor-Arginine (nor-NOHA, Bachem, Torrance, CA – 250 µg/mouse), sildenafil (Sigma-Aldrich, 0.001, 0.01 and 0.1 mg/mouse), NaNO_2_ (Sigma-Aldrich, 0.72 mg/mouse), and selected combinations of these drugs via IP, twice a day starting on day 0. All drugs were diluted in saline and a total volume of 100 µl/mouse per dose was injected per treatment. L-arginine dosing was based in a previous human malaria study showing that a similar dose (12 g, or approximately 200 mg/kg) improved endothelial function in patients with moderately severe falciparum malaria [19]. In healthy rats, intravenous injection of L-arginine induces a rapid and transient (60 minutes) increase in plasma levels, but arginine levels in the brain remain elevated for more than 8 hours [38], [39]. Oral supplementation with 10 mg/kg/day of BH4 prevents endothelial dysfunction in murine models of non malarial pathologies with chronic vascular oxidative stress [40], [41], but doses as high as 50 mg/Kg IP twice a day were used and well tolerated [42]. As administration of BH4 in the presence of conditions with increased oxidative stress may lead to its rapid oxidative degradation and thus limited duration of the beneficial effects [43], we used a high dose in our experiments. Nor-NOHA is a potent arginase inhibitor and the dosage was based in a previous study showing that a similar dose increased cellular L-arginine content in lungs of ovalbumin-exposed mice [44]. Sildenafil dosing was based on studies showing that similar doses given orally or subcutaneously improved recovery after stroke in rats [45], [46]. NaNO_2_ dose was calculated to release, in the presence of deoxyhemoglobin, potentially the same amount of NO as 1 mg of DPTA-NO using the following reaction: NO_2_^−^ (nitrite)+HbFe^2+^ (deoxyhemoglobin)+H^+^→HbFe^3+^ (methemoglobin)+NO+OH^−^ [34].

### Preparation and implantation of Alzet osmotic pumps

We also used an alternative method of L-arginine delivery using osmotic pumps (Alzet, Cupertino, CA) to achieve a continuous delivery of 200 mg/kg/day of L-arginine during the first 6 days of infection. Osmotic pumps (model 1003D, constant delivery rate of 1 µL/hour for 3 days) were filled with the appropriate solution (160 mg/ml of arginine or saline as control – 100 µL final volume) and primed in 0.9% sterile saline at 37°C for approximately 4 hours to ensure immediate delivery of the contents after implantation. Mice were anesthetized using isofluorane and the primed pumps were implanted subcutaneously on the back, slightly posterior to the scapulae, under sterile conditions in the same day of infection. Pumps were changed on day 3 post infection. A bolus injection of L-arginine (4 mg/mouse) or saline was given IP just after implantation to a rapid establishment of a steady state condition.

### Hematocrit, plasma nitrite and exhaled NO

Hematocrit levels were measured in blood samples (20 µl) on day 6 post infection using heparinized micro-hematocrit capillary tubes (Chase, Rockwood, TN). Plasma nitrite content was measured using an ENO-20 NOx Analyzer (Eicom, San Diego, CA) according to the manufacturer's instructions. Plasma samples (20 µl) collected on day 6 twelve hours after the previous dose of any given treatment were mixed with the same volume of methanol using a vortex for 10 sec, centrifuged at 10,000 G for 10 min and the supernatant was collected and frozen (−80°C) until reading. Levels of exhaled NO were measured using a NOA 280i NO analyzer (Sievers, Boulder, CO) one hour after the morning treatment on day 5 post infection. Mice were placed in a custom plexiglas chamber for one min and, after this period, the NO content of one sample of the chamber's air was measured.

### Cardiovascular parameters

Remote measurement of heart rate and blood pressures (systolic, diastolic, pulse, and mean arterial pressures) was made with Data System International telemetry devices (DSI, St. Paul, MN) on individually housed mice at room temperature as previously described [47]. During sterile surgery, the catheter tip of a TA11PA-C20 unit was inserted into the carotid artery of the anesthetized mouse (xylazine 10 mg/Kg and ketamine 150 mg/Kg, IP) and the transmitter was secured in a subcutaneous pocket in the dorsal neck region. Mice were allowed to recover for at least 5 days post surgery and all measurements were carried out in the home-cage environment. Data were collected during a 30 min baseline period after which an IP injection of the drug was given and parameters were followed during the next hour (experimental period). During the baseline and experimental periods mice were left alone in the procedure room providing a noise free environment. Heart rate, systolic, diastolic, and pulse pressures data were averaged as 10 seconds bins for each animal and the average for each bin from the same time point in each treatment group was determined. Means from bins representing the first 30 minutes before injection were then averaged to calculate a baseline for each group. Data from each bin was converted and plotted as percentage of baseline.

### Statistical analysis

**Figure 1:**
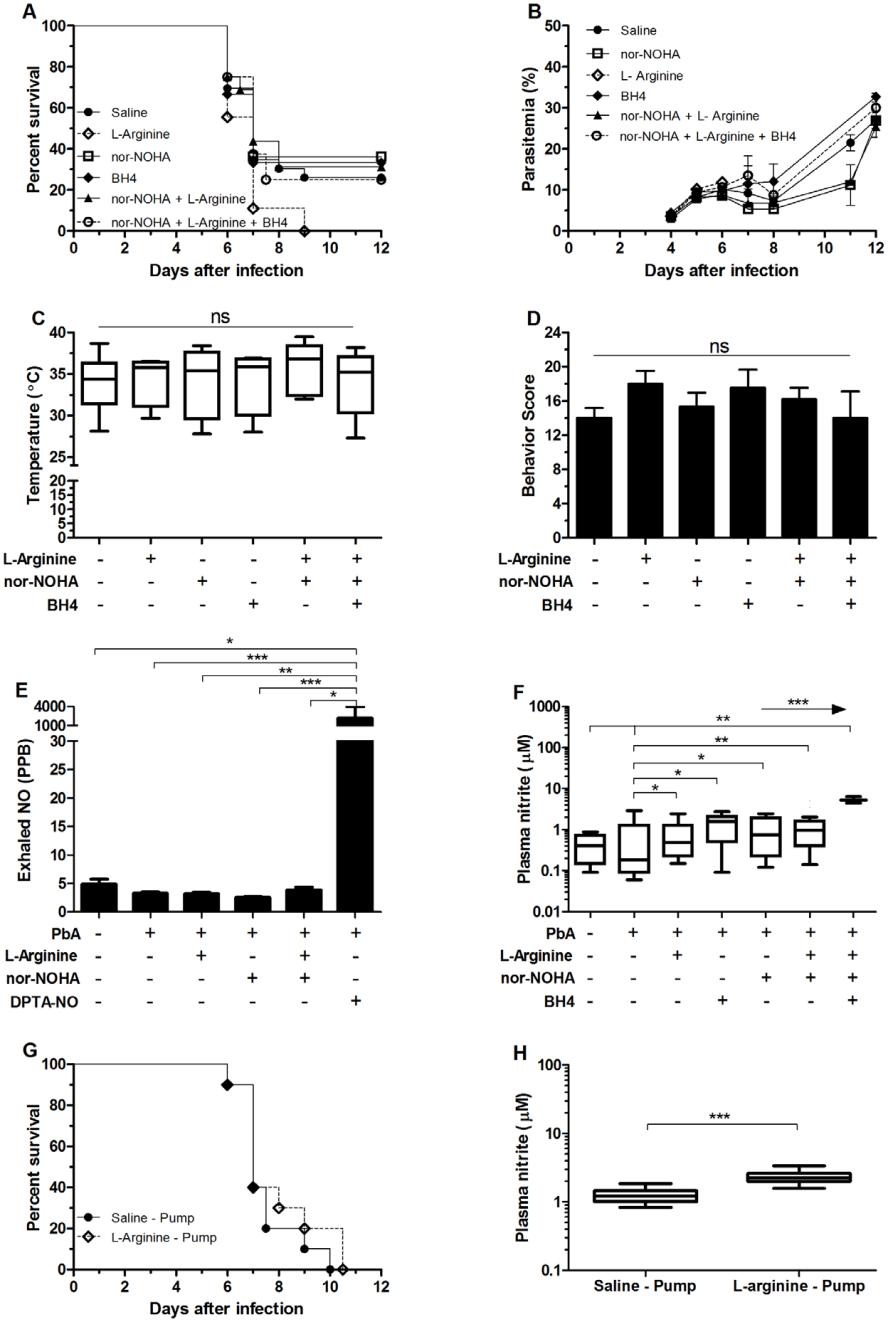
L-arginine and/or tetrahydro-L-Biopterin (BH4) supplementation combined or not with arginase inhibition did not prevent ECM. Cumulative survival (A), course of parasitemia (B), rectal temperature (C), and motor behavior score (D) of PbA-infected mice treated with bolus injections of saline (n = 22), L-arginine 4 mg/mouse (n = 9), the arginase inhibitor Nω-hydroxy-nor-Arginine (nor-NOHA) 250 µg/mouse (n = 15), BH4 1 mg/mouse (n = 9), nor-NOHA+L-arginine (n = 16), and nor-NOHA+L-arginine+BH4 (n = 9). Rectal temperature and motor behavior score were measured on day 6 of infection. Exhaled NO (E) from selected groups was measured 1 hour after the morning treatment on day 5 of infection (n≥5 per group). Plasma nitrite (F) from selected groups treated with bolus injections. Cumulative survival (**G**) and plasma nitrite (**H**) of PbA-infected mice prophylactic treated with continuous L-arginine or saline supplementation using implanted osmotic pumps (n = 10 per group). Plasma nitrite (F, H) was measured on samples collected prior to the morning dosing on day 6 of infection (n≥5 per group). *p<0.05, **p<0.01, ***p<0.001, arrows indicate the presence of a linear trend.

**Figure 2:**
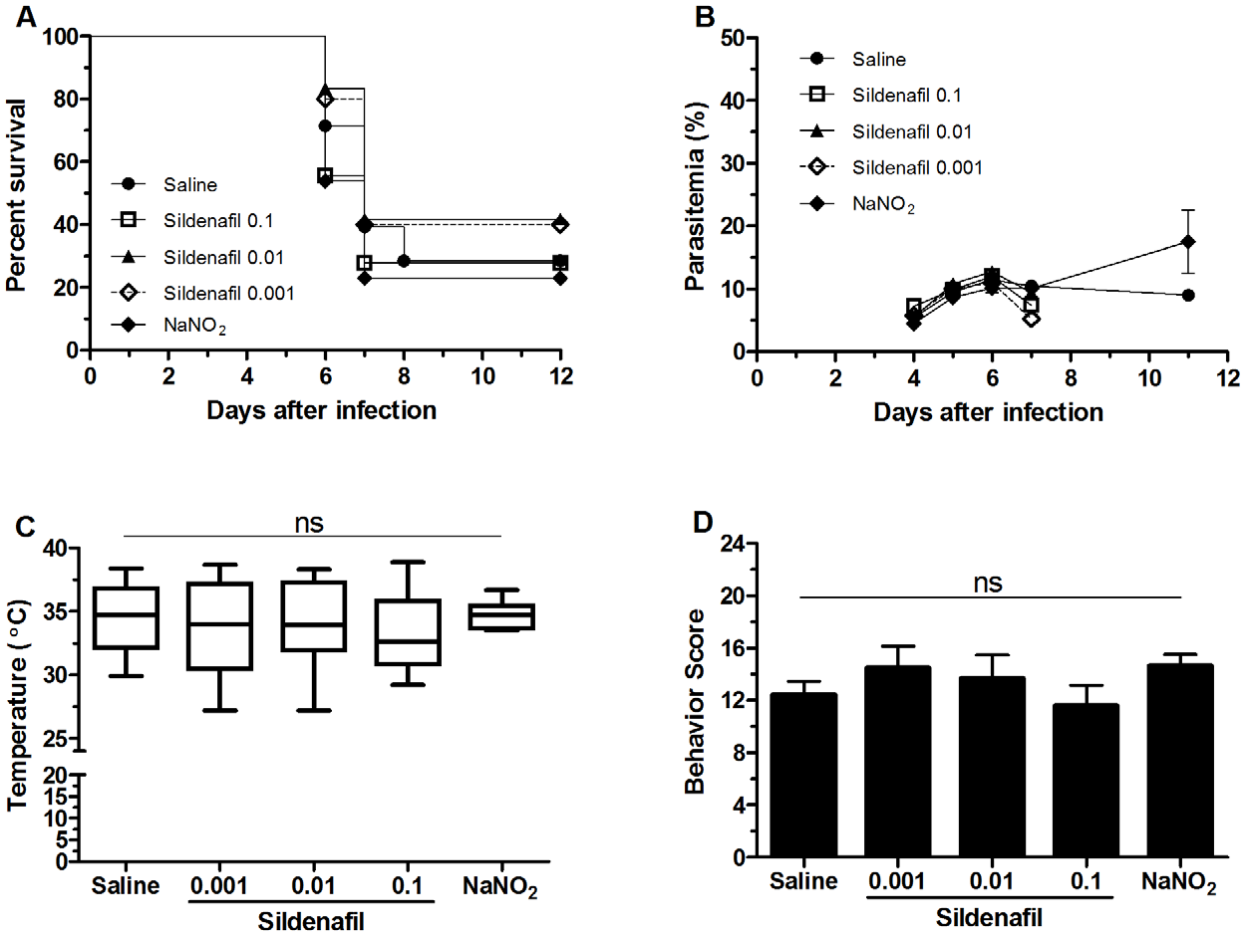
PDE-5 inhibition with sildenafil or sodium nitrite (NaNO_2_) supplementation did not prevent ECM. Cumulative survival (A), course of parasitemia (B), rectal temperature (C), and motor behavior score (D) of PbA-infected mice prophylactic treated with saline (n = 28), NaNO_2_ (n = 13), and sildenafil at 0.1 (n = 19), 0.01 (n = 12) or 0.001(n = 5) mg/mouse. Rectal temperature and motor behavior score were measured on day 6 of infection. There were no significant differences in the parameters analyzed (p>0.05 for all comparisons).

**Figure 3:**
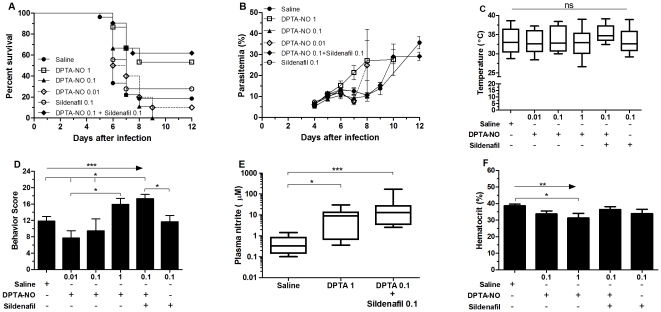
PDE-5 inhibition with sildenafil decreases the amount of dipropylenetriamine NONOate (DPTA-NO) necessary to prevent ECM. Cumulative survival (A), course of parasitemia (B), rectal temperature (C), and motor behavior score (D) of PbA-infected mice treated with saline (n = 29), DPTA-NO (1, 0.1 or 0.01 mg/mouse, n = 10 per group), DPTA-NO+sildenafil (0.1 mg/mouse of each drug, n = 18), and sildenafil (0.1 mg/mouse, n = 19). Plasma nitrite (E) and hematocrit (F) from selected groups were measured on samples collected prior to the morning dosing on day 6 of infection (n≥5 per group). *p<0.05, **p<0.01, ***p<0.001, arrows indicate the presence of a linear trend.

**Figure 4:**
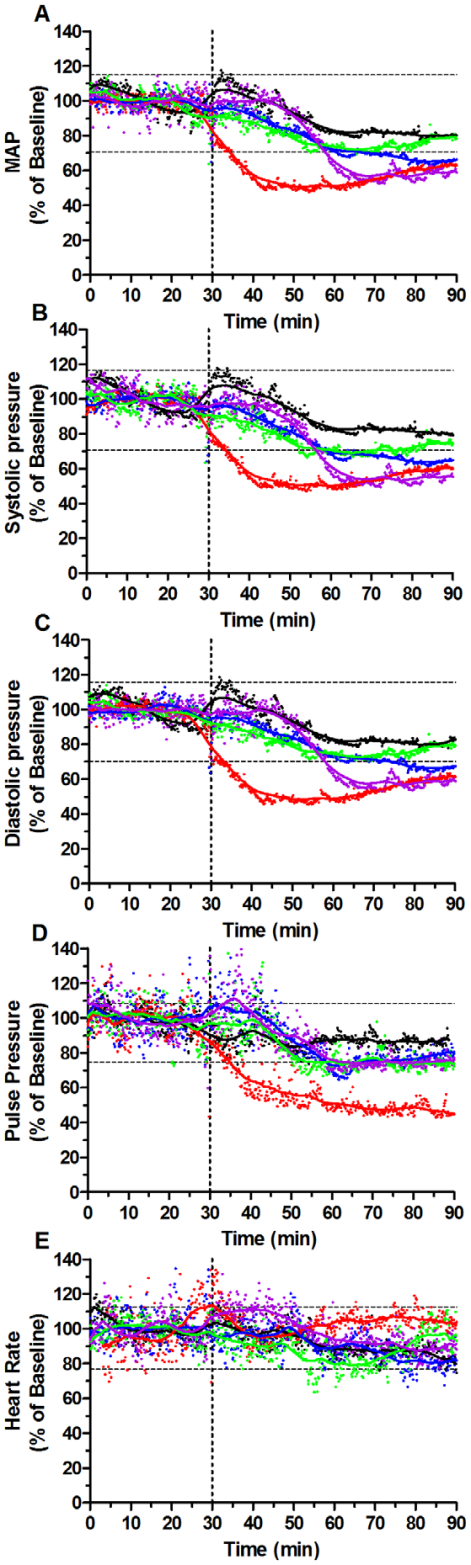
PDE-5 inhibition with sildenafil does not increase dipropylenetriamine NONOate (DPTA-NO) side-effects in cardiovascular parameters. Changes in mean arterial pressure (MAP, A), systolic pressure (B), diastolic pressure (C), pulse pressure (D) and heart rate (E) following one IP injection of saline (black dots and lines), DPTA-NO (1 and 0.1 mg/mouse, red and blue dots and lines, respectively), sildenafil (0.1 mg/mouse, green dots and lines), and DPTA-NO+sildenafil (0.1 mg/mouse of each drug, purple dots and lines). Vertical doted lines represent the time when the IP injection was given and separate the baseline period from the experimental period. Results are expressed as the percentage change in relation to the mean of the baseline period for each group. Horizontal doted lines represent the range of values falling within the mean plus and minus two standard deviations (SD) of the baseline value calculated for saline treated group. n = 4 per group.

Results were expressed as means and standard errors of the mean unless otherwise stated. The log-rank test was used to compare the different survival curves (Figures 1A, 1G, 2A, and 3A). Two-way repeated measurement ANOVA with Bonferroni posttests was used to analyze parasitemia curves (Figures 1B, 2B, and 3B). Kruskal-Wallis test with Dunn's Multiple Comparison post tests were used when comparing if one parameter varied among three or more different treatment groups (Figures 1C–1F, 2C–2D, and 3C–3F). Mann-Whitney test was used when just two groups were analyzed (Figures 1H). When combined prophylactic treatments and multiple concentrations of DPTA-NO or sildenafil were tested, post-tests to check for linear trend following Kruskal-Wallis test were also performed (Figures 1F, 3D and 3F). A p-value<0.05 was considered significant. Lowess curves were calculated for the cardiovascular parameters to show the trend of the data in each group. Normal ranges for each parameter were calculated based in values obtained from four saline treated animals. The range of values falling within the mean plus and minus two standard deviations (SD) was considered normal and treatments that decrease or increase the parameter values outside this range were considered to affect the parameter (Figures 4A–4E). All statistics were calculated using GraphPad Prism 4.01 (GraphPad Software, San Diego, CA).

## Results

### Strategies to increase L-arginine availability or improve NOS activity did not prevent ECM

We first attempted to prevent ECM in PbA-infected mice by addressing the possibility that low NO bioavailability is related to hypoargininemia, whether or not caused by increased arginase activity, or NOS dysfunction caused by BH4 deficiency. The strategies consisted of supplementing arginine and/or BH4, with or without inhibition of arginase. However, none of these prophylactic treatments, given alone or in combination, were able to decrease mortality in PbA-infected mice in the doses and schedules used (Figure 1A). The prophylactic treatments did not change the course of parasitemia (Figure 1B) or the clinical status of the mice during infection, as verified by rectal temperature (Figure 1C) and motor behavior (Figure 1D) on day 6 of infection.

We determined the levels of exhaled NO in mice of groups that received L-arginine, nor-NOHA or the combination of L-arginine+nor-NOHA, but no significant changes in exhaled NO levels were observed when compared with uninfected controls or animals prophylactically treated with saline (Figure 1E). Comparatively, mice that received 1 mg of DPTA-NO showed very high levels of exhaled NO (Figure 1E). Although exhaled NO level measurements in mice can detect substantial changes in NO bioavailability, it may not be sensitive enough to detect more subtle variations as well as increases in cumulative NO production [48]. Therefore, we measured plasma nitrite levels on day 6 post infection. Mean plasma nitrite levels in all groups were significantly higher than in the saline group (Figure 1F), indicating that NO production was improved by these prophylactic treatments. Mean plasma nitrite levels of mice prophylactically treated with L-arginine+nor-NOHA+BH4 was higher than those of mice that received nor-NOHA+L-arginine and the latter was higher than those of mice that received only nor-NOHA. This trend was statistically significant (Figure 1F) indicating that the treatments had an additive effect. However, these subtle increases in NO availability were not sufficient to prevent the development of ECM.

Failure to keep plasma concentrations of L-arginine constantly above a therapeutic threshold during the course of infection could explain the inefficacy of the protocol of L-arginine supplementation used in preventing ECM. Nonetheless, supplementation of L-arginine with the same daily dose used for bolus injection given continuously using osmotic pumps did not improve survival (Figure 1G), incidence of ECM, course of parasitemia, or clinical status (verified by rectal temperature and motor behavior) of PbA-infected mice when compared to infected control mice. Plasma nitrite levels on day 6 post infection were significantly higher in L-arginine supplemented mice than saline controls as revealed when comparing absolute values (Figure 1H) and the delta nitrite (difference between the day 6 and baseline – day 0 – values). Mean baseline nitrite concentrations in plasma were not different between saline and L-arginine supplemented groups.

### Sodium nitrite or sildenafil prophylactic treatments do not prevent ECM

It has been experimentally [31], [32] and clinically [33], [34] shown that deoxygenated hemoglobin in an acidic environment reduces nitrite to NO causing vasodilation. This effect may have an important function in providing NO in sites of low oxygenation, such as in ischemia. As ECM mice present vasospasm and regional deficits of blood flow in the brain and their adjunctive treatment with a vasodilator is capable of improving prognosis [14], we reasoned that prophylactic NaNO_2_ administration would be able to generate NO in such ischemic sites ameliorating microcirculatory parameters and decreasing mortality. However, there was no significant difference in mortality and parasitemia between mice that received or not NaNO_2_ (Figure 2A–2B), nor in rectal temperature or motor behavior (Figure 2C–2D), showing that the prophylactic treatment did not prevent ECM.

Sildenafil increases brain cGMP levels through inhibition of PDE-5 [30], providing a method to optimize the L-arginine-NOS-NO pathway so that smaller amounts of NO can still have proper physiological effects. Based on this premise, we tested whether the prophylactic treatment with sildenafil could prevent the development of ECM. However, even at higher doses (0.1 mg/mouse), sildenafil did not change survival, course of parasitemia, rectal temperature, or motor behavior (Figure 2A–2D).

### Combined sildenafil administration markedly reduces the amount of exogenous NO needed to prevent ECM

When given at the effective dose against ECM, DPTA-NO causes marked hypotension and decreases hematocrit in mice [15], [16]. We asked whether prophylactic treatment with DPTA-NO at much lower amounts (0.1 and 0.01 mg/mouse every 12 hours) could also be effective in preventing ECM having lower side effects. This hypothesis was not confirmed, as while prophylactic DPTA-NO treatment at 1 mg/mouse decreased mortality and improved the behavior score on day 6 without significantly changing the course of parasitemia, no significant differences in survival, course of parasitemia, temperature and motor behavior were observed between mice prophylactically treated with lower doses of DPTA-NO and mice receiving saline (Figures 3A–3D). However, prophylactic treatment with a lower dose (0.1 mg/mouse) of DPTA-NO in combination with sildenafil at 0.1 mg/mouse decreased mortality and improved motor behavior to similar levels as observed with DPTA-NO at 1 mg/mouse without significant changes in the course of parasitemia or rectal temperature (Figures 3A–3D). Mice that received the survival-improving doses of DPTA-NO (1 mg) or DPTA-NO 0.1 mg plus sildenafil (0.1 mg) presented high plasma nitrite levels when compared with animals that received saline (Figure 3E), showing an increase in the NO bioavailability in these groups. The use of lower amounts of DPTA-NO allowed by the addition of sildenafil also resulted in the avoidance of the adverse effect of high DPTA-NO doses on hematocrit (Figure 3F).

### Lower amounts of exogenous NO combined with sildenafil decrease the cardiovascular side-effects observed with high doses of DPTA-NO

We evaluated whether the effects of DPTA-NO injection on arterial pressure (systolic, diastolic, pulse and MAP) as well as on heart rate would be avoided or minimized by decreasing the dose in the presence or not of sildenafil (0.1 mg). Administration of DPTA-NO 1 mg decreased MAP, systolic and diastolic pressure levels to around 50% of baseline 10 minutes after injection and these parameters were kept low during the observation period (Figure 4A–4D). Interestingly, the pulse pressure decrease (defined as systolic minus diastolic pressure) shows that the drug had a greater effect on systolic than on diastolic pressure. Mice that received DPTA-NO 0.1 mg, or sildenafil 0.1 mg/mouse showed a less intense variation in these parameters, which were kept mostly within the range observed in mice receiving only saline, or showed a late decrease (Figure 4A–4D). Mice that received DPTA-NO 0.1 mg plus sildenafil 0.1 mg/mouse showed a delayed decrease in systolic, diastolic, and mean arterial pressures, but pulse pressure was kept within the normal range during all the observation period (Figure 4A–4D). There were no significant differences in heart rate curves of mice in the different groups and all of them were kept within the normal range (Figure 4E).

## Discussion

Low NO bioavailability has been implicated in the pathogenesis of both human and experimental CM [15], [16], [20], [49]. In the present study, we show that in ECM: a) prophylactic therapies aiming to increase the amount of L-arginine available for NOS through L-arginine or BH4 supplementation, with or without arginase inhibition, in single or combined therapies, increased NO bioavailability but did not prevent ECM in the doses and schemes used; b) nitrite supplementation or PDE-5 inhibition also did not prevent ECM in the doses and schemes used; c) a high amount of NO supplementation provided by a potent NO-donor is necessary to prevent the disease, but this amount can be reduced when combined with a PDE-5 inhibitor; d) the combined sildenafil+DPTA-NO (0.1 mg of each drug) prophylactic therapy did not present all the adverse effects observed in the prophylactic treatment with the high dose of DPTA-NO alone.

There are numerous potential explanations for the failure of the NO-promoting strategies used in this study in preventing ECM. One consideration is that the prophylactic agents were not administered in sufficient amounts to achieve therapeutic levels during a sufficient period. The doses chosen for L-arginine, nor-NOHA and BH4 were similar or even higher than those used in previous studies showing their pharmacodynamic effects on relevant parameters such as reversal of endothelial dysfunction in human malaria (L-arginine, [19]) and vascular oxidative stress (BH4, [40], [41]) or arginase inhibition in a model of asthma (nor-NOHA, [44]). However, we have not performed pharmacokinetic studies of the compounds we used. In normal rats intravenous injection of 0.1 mmol of L-arginine (57 mg/kg) induces a transient (60 minutes) increase in plasma levels but sustained (more than 8 hours) increase in brain levels [38], [39]. In addition, DPTA-NO prevents ECM despite its short period (less than four hours) in the bloodstream after IP injection [16], indicating that it is not necessary to keep high levels of NO during long periods of time to prevent ECM. On the other hand, pharmacokinetics varies among species and malaria infection can change the pharmacokinetic profile of compounds as it has been shown for L-arginine [50].

The fact that the injection of L-arginine, BH4 and/or nor-NOHA did not result in increased levels of exhaled NO might suggest in principle that the compounds were not properly absorbed or these doses were insufficient to induce plasma levels and tissue distribution compatible with the generation of detectable levels of NO. This concern is particularly relevant for L-arginine since a similar dose given to patients with severe malaria was able to increase exhaled NO levels by 55% [19]. However, there are a number of factors to consider: 1) methodologically, the volume of exhaled air and its NO content are much higher in humans facilitating detection of relatively small differences between groups [51]. In mice, although exhaled NO level measurements can detect marked changes in NO bioavailability (e.g., after DPTA-NO injection), it may not be sensitive enough to detect more subtle variations as well as increases in cumulative NO production [48]; 2) Timing is important. In the study by Yeo et al. [19], exhaled NO was measured at a single point at the end of the 30-minute L-arginine infusion and detected a mean 55% increase in exhaled NO, but it is not known for how long these levels were maintained. In mice, we measured exhaled NO levels one hour after an intraperitoneal bolus injection and because the baseline levels in mice were already low (3–5 PPB), small and/or transient increases might be harder to detect; 3) The increase in exhaled NO after L-arginine infusion was obtained in patients with moderately severe malaria, whose plasma levels of NO-quenching acellular hemoglobin is high but yet less than half those seen in patients with severe malaria (2.6 versus 5.4 uM, respectively - [19]). It is not know whether L-arginine infusion would have the same effect in the latter group since free hemoglobin stoichiometrically consumes micromolar quantities of NO [52], [53]. In mice, mean acellular plasma hemoglobin levels also increase with disease severity (plasma levels of about 3 uM on day 4 and 7 uM on day 6 of infection - [16]).

For these reasons, plasma nitrite levels are likely to be a more reliable indicator of the efficacy of compound delivery. Indeed, all prophylactically treated groups (L-arginine when given in bolus or continuous delivery, and bolus injections of nor-NOHA, BH4 and the combinations) showed increased plasma nitrite levels compared to PbA-infected mice receiving saline (Figures 1F and 1H). Moreover, treatments were additive, with a significant trend for higher nitrite levels in groups receiving two (L-arginine+nor-NOHA) or three (L-arginine+nor-NOHA+BH4) compounds. In addition, it is important to stress that the plasma nitrite measurements in groups treated with bolus injection were made 12 hours after dosing (day 6 in the morning, dose received on day 5 in the evening), therefore showing that these are not just transient increases in nitrite levels, but correspond to lasting increased production of NO. Therefore, we may conclude that the bolus injections of L-arginine and/or nor-NOHA combined or not with BH4 supplementation, resulted in increased NO bioavailability, but this was not enough to prevent ECM. Indeed, plasma nitrite levels achieved by L-arginine and/or BH4 supplementation, with or without arginase inhibition, were increased but not to same degree as those attained by prophylactic treatment with DPTA-NO or DPTA-NO plus sildenafil (see Figure 3E).

The half-life of L-arginine is shorter in patients with malaria when compared to healthy adults [50] and a similar situation could occur in PbA infected mice, which could explain the inefficacy of the L-arginine prophylactic treatment to prevent ECM. We tried to accomplish enhanced NO production using an improved method for L-arginine delivery with subcutaneous osmotic pumps, which allow its continuous infusion. Although this method also resulted in increased plasma nitrite levels on day 6 of infection (Figure 1H) this scheme did not prevent ECM, suggesting that the fact that animals were supplemented with bolus injection was not the single factor responsible for the inefficacy of L-arginine supplementation in preventing ECM. Nevertheless, we do not discard the possibility that higher doses of L-arginine, with or without association with other compounds, could be effective to generate the high levels of NO needed to prevent ECM.

Other circumstances can be claimed to explain the failure of most treatments to prevent ECM. L-arginine is used as a substrate by five different sets of enzymes: a) arginyl-tRNA synthetase; b) NO synthases; c) arginases; d) arginine:glycine amidinotransferase; and e) arginine decarboxylase [23]. We showed that it is possible to increase the production of nitrite, indicative of NOS activity, during the infection by the treatment with a non-selective arginase inhibitor associated or not with L-arginine supplementation. This indicates that a deficiency of L-arginine due to arginase consumption occurs in ECM, but the possible indirect consumption of L-arginine by the other three pathways was not ruled out. Of particular interest is the enzyme arginine decarboxylase, which synthesizes agmatine, an endogenous neuromodulator induced in response to stress and/or inflammation, that increases the expression of eNOS, irreversibly inhibits nNOS, and downregulates iNOS [54], [55], [56]. It remains to be shown whether a potential increase in activity of such enzymes interferes with the total NO generation in ECM. Intracellular L-arginine transport through the cationic amino acid transporters can also regulate substrate availability for NOS [24]. Downregulation of these transporters during the infection also could explain the failures in the treatments.

NOS activity is inhibited in ECM [26], but the mechanism of inhibition is unknown. We hypothesized that hypoargininemia due to L-arginine consumption by arginase and/or BH4 deficiency might play a role in NOS malfunction in ECM. The fact that the combined injection of L-arginine+nor-NOHA+BH4 induced higher levels of plasma nitrite than L-arginine+nor-NOHA and that these two combinations induced levels higher than each compound separately suggests that increased arginase activity and eNOS uncoupling both contribute to impaired NO production in ECM. Regulation of NOS is complex and dependent on several co-enzymes (such as NADPH, flavin adenine dinucleotide and flavin mononucleotide) and co-factors (BH4, calmodulin, heme, and calcium) and their activity can be regulated by several kinases, phosphatases and the endogenous competitive inhibitor asymmetrical dimethylarginine (ADMA) [48], [57]. Indeed, plasma levels of ADMA are increased in patients with severe malaria when compared to healthy controls and were an independent predictor of mortality in these patients [22]. The plasma L-arginine/ADMA level ratio has been considered an important factor leading to eNOS inhibition in malaria and sepsis [22], [58]. In ECM, plasma ADMA levels have been shown to decrease instead of increase [16], which may be considered an intrinsic difference between HCM and the murine model. However, the plasma L-arginine/ADMA level ratio is also decreased in ECM and this measure may be more important in determining NOS inhibition by ADMA than its absolute plasma levels. Therefore, deficiency of other co-enzymes and co-factors, decrease of L-arginine/ADMA ratio during infection or mechanisms involving kinases and phosphatases can also be involved in the phenomenon.

The failure of sildenafil alone in preventing ECM may as well be due to insufficient amounts being delivered or could be explained based on data from erectile dysfunction studies showing that PDE-5 inhibitors have no effect when the concentrations of NO and cGMP are very low [29], [59], which is the case of mice presenting ECM [16]. This last interpretation is in line with our demonstration that when co-administered with an NO-donor sildenafil shows a protective effect against ECM development. In this sense, the amount of exogenous NO provided by the injection of 0.1 mg of DPTA-NO would generate the critical signaling through cGMP formation which by itself, at this concentration, would be insufficient to prevent ECM, but became effective with the extended potency provided by PDE-5 inhibition. The synergism between the two drugs is also apparent regarding their effects on cardiovascular parameters, since a late decrease in blood pressure was observed with the drug combination but not with each drug independently at the same concentrations. The finding that the combination of lower doses of DPTA-NO with sildenafil is able to increase NO bioavailability as shown by increased plasma nitrite levels and to decrease ECM incidence brings new prospects for the use of NO donors in severe malaria, the combination being an alternative to reduce the potent side effects secondary to the injection of large amounts of DPTA-NO. Although it still affected blood pressure, there was a considerable delay in relation to DPTA-NO alone at high dose. In addition, the lower dose of DPTA-NO, with or without sildenafil, prevented its deleterious effects on hematocrit. It is therefore possible to envisage manipulations in the treatment strategy to further minimize its cardiovascular effects (e.g., administering first the DPTA-NO at low dose with a later dose of sildenafil to potentiate the low amounts of NO still present after some time of DPTA-NO administration).

We also showed that the treatment with sodium nitrite did not prevent ECM. The rationale for this treatment was based on studies showing that nitrite would function as an “on demand” NO donor in inflammatory compromised brain vessels that would present low pH and O_2_ concentration [31], [32], [33], [34]. Although these features are present in ECM, if NO generation from sodium nitrite oxidation occurred, it was not sufficient to modify the course of the disease.

In summary, the present study indicates that prophylactic strategies based on NO restoration to improve the outcome of ECM may be more complicated than originally envisaged. L-arginine supplementation alone or associated with secondary strategies such as arginase blockade and BH4 supplementation were able to increase NO bioavailability but were shown to be ineffective in preventing ECM in the doses and schemes used. Therefore, more research is necessary to determine not only the efficacy of alternative dosing and delivery systems but also to better characterize the effect of interventions on relevant readouts such as NO generation, endothelial function and activity of NOS isoforms especially in the brain. On the other hand, our results with the combination DPTA-NO at lower doses with sildenafil indicates that NO-based prophylactic treatments can be optimized to decrease potential side effects caused by the administration of high doses of NO.
